# Supplementary material for: Discovery of fungal oligosaccharide-oxidising flavo-enzymes with previously unknown substrates, redox-activity profiles and interplay with LPMOs
Source: Nat Commun. 2021 Apr 9;12:2132. doi: 10.1038/s41467-021-22372-0 (PMC8035211; doi:10.1038/s41467-021-22372-0)
Supplement: Supplementary file 1 — Supplementary Information [file 41467_2021_22372_MOESM1_ESM.pdf]

Supporting Information

Functional diversity analyses of fungal oligosaccharide-oxidising flavo-enzymes reveal direct activation of LPMOs

Majid Haddad Momeni[1,2], Folmer Fredslund[3], Bastien Bissaro[2], Olanrewaju Raji[4], Thu V. Vuong[4], Sebastian Meier[5], Tine Sofie Nielsen, Vincent Lombard[6], Bruno Giugliarelli[7], Frédéric Biaso[7], Mireille Haon[2], Sacha Grisel[2], Bernard Henrissat[6], Hededam Welner [3], Emma R. Master[4,8], Jean-Guy Berrin[2*], Maher Abou Hachem[1*]

[1]Department of Biotechnology and Biomedicine, Technical University of Denmark, Søltofts Plad, 2800 Kgs. Lyngby, Denmark

[2]INRA, Aix Marseille Université, Biodiversit_e et Biotechnologie Fongiques (BBF), UMR1163, F-13009 Marseille, France

[3]The Novo Nordisk Center for Biosustainability, Kemitorvet, building 220, DK-2800 Lyngby, Denmark

[4]Department of Chemical Engineering and Applied Chemistry, University of Toronto, Toronto, ON, Canada

[5]Department of Chemistry, Technical University of Denmark, Kemitorvet, 2800 Kgs Lyngby, Denmark

[6]Architecture et Fonction des Macromolécules Biologique, UMR 7257 CNRS, USC 1408, Aix Marseille Université, Marseille, France

[7]Aix-Marseille Université, CNRS, UMR7281 Unité de Bioénergétique et Ingénierie des Protéines (BIP), F-13402 Marseille, France

[8]INRAE, USC1408 Architecture et Fonction des Macromolécules Biologiques (AFMB), Marseille, France

[9]Department of Biological Sciences, King Abdulaziz University, Jeddah, Saudi Arabia

[10] Department of Bioproducts and Biosystems, Aalto University; FI-00076 Aalto, Kemistintie 1, Espoo, Finland

Correspondence: Maher Abou Hachem (maha@bio.dtu.dk) and Jean-Guy Berrin (jean-guy.berrin@inrae.fr)

Table of content:

Supplementary Table 1. Sequences of AA7 enzymes selected for recombinant expression.
Supplementary Table 2. Substrate panel for screening AA7 enzymes.
Supplementary Table 3. Apparent kinetic parameters of *Pb*Chi7A towards less-preferred substrates.
Supplementary Table 4. Data collection and refinement statistics for *Fg*CelDH7C and *Fg*Chi7B.

Supplementary Fig. 1. Oxidase and dehydrogenase activity screening of AA7 enzymes.
Supplementary Fig. 2. The activity-pH profiles of AA7 enzymes and thermal stability of *Fg*CelDH7C.
Supplementary Fig. 3. NMR assignment spectra of GalNAc, GlcNAc and GNB.
Supplementary Fig. 4. Time course NMR analyses of GalNAc and GlcNAc oxidation by *Mo*Chi7A.
Supplementary Fig. 5. NMR assignment spectra of cellobiose oxidation by *Fg*CelDH7C.
Supplementary Fig. 6. NMR time course analysis of cellobiose oxidation by *Fg*CelDH7C.
Supplementary Fig. 7. The dependence of cellooligosaccharide oxidation by *Fg*CelDH7C on DCIP.
Supplementary Fig. 8. Electron density in the active site of *Fg*Chi7B and *Fg*CelDH7C
Supplementary Fig. 9. Structural domains and comparisons of AA7 active site loops.
Supplementary Fig. 10. Active site signatures of AA7 phylogenetic clades.
Supplementary Fig. 11. Surface electrostatic comparison of AA7 structures.
Supplementary Fig. 12. PASC degradation assay with *Fg*CelDH7C and *Pa*LPMO9E or *Pa*LPMO9H.
Supplementary Fig. 13. Avicel degradation assay with *Fg*CelDH7C-*Pa*LPMO9H.
Supplementary Fig. 14. Avicel degradation assay with *Fg*CelDH7C-*Ls*AA9A.
S**upplementary Fig. 15.** Avicel degradation assay with *Fg*CelDH7C or *Pa*CDH and *Pa*LPMO9H.
Supplementary Fig. 16. Avicel degradation assay with *Pa*LPMO9H and either *Fg*CelDH7C or *Mo*Chi7A.
Supplementary Fig. 17. Structural signature of AA7 activity on C2 *N*-acetylated oligosaccharides.

Supplementary Table 1. AA7 sequences selected for recombinant expression.

Organism	GenPept accession number	Name	Clade	Size of the recombinant enzymes	MW* (Da)	$\varepsilon^*_{280\ nm}$ ($M^{-1}\ cm^{-1}$)
Aspergillus nidulans	XP_660252	*An*AA7A	I	551(549)	59691.6	112690
Fusarium graminearum	XP_011319890	*Fg*CelDH7C	IIa	488(476)	53225.1	85955
Fusarium graminearum	CEF79461	*Fg*Chi7B	Va	486(484)	53245.2	81040
Magnaporthe oryzae	XP_003717634	*Mo*Chi7A	Vb	704(698)	72202.9	77375
Polyporus brumalis	RDX44700.1	*Pb*Chi7A	VI	514(510)	52380.8	78395

All selected AA7s were predicted to be secreted using SignalP 5.0 (http://www.cbs.dtu.dk/services/SignalP/). The recombinant enzymes were produced without their native signal peptides and as C-terminal fusions to an "AAAHHHHHH" purification tag. The number in parenthesis is the size of the native mature peptide.

*The indicated molecular masses and molar extinction coefficients for the recombinant enzymes are determined with the ProtParam tool (https://web.expasy.org/protparam/).

Supplementary Table 2. Substrate panel for the activity screening of AA7 enzymes.

	Substrate	Chemical name or abbreviation
Monosaccharides	1. Glucose	Glc
	2. Galactose	Gal
	3. Mannose	Man
	4. Xylose	Xyl
	5. Glucosamine	GlcN
	6. Galactosamine	GalN
	7. L-Arabinose	Ara
	8. Fructose	Fru
	9. *N*-Acetyl glucosamine	GlcNAc
	10. *N*-Acetyl galactosamine	GalNAc
	11. *N*-Acetyl mannosamine	ManNAc
Disaccharides	12. Cellobiose	Glc-(β1→4)-Glc
	13. Gentiobiose	Glc-(β1→6)-Glc
	14. Sophorose	Glc-(β1→2)-Glc
	15. Lactose	Gal-(β1→4)-Glc
	16. Xylobiose	Xyl-(β1→4)-**D**-Xyl
	17. Lacto-*N*-biose (LNB)#	Gal-(β1→3)-GlcNAc
	18. Galacto-*N*-biose (GLB)#	Gal-(β1→3)-GalNAc
	19. Chitobiose*	GlcNAc-(β1→4)-GlcNAc
	20. Trehalose	Glc-(α1→1)α-Glc
	21. Sucrose	Glc-(α1→2)-β-Fru
	22. Turanose	Glc-(α1→3)-β-Fru
	23. Maltose	Glc-(α1→4)-Glc
	24. Melibiose	Gal-(α1→6)-Glc
	25. Mannobiose#	Man-(α1→2)-Man
Oligosaccharides	26. Melezitose	Glc-(α1→3)Fru*f*-(β2→1α)-Glc
	27. Panose	Glc-(α1→6)-Glc(α1→4)-Glc
	28. Raffinose	Gal(α1→6)-Glc(α1→2β)-Fru
	29. Arabinotriose*	L-Ara*f*-(α1→5)-L-Ara*f*(α1→5)-L-Ara
	30. Chitotriose*	GlcNAc(β1→4)-GlcNAc(β1→4)-GlcNAc
	31. Chitotetraose*	GlcNAc(β1→4)-GlcNAc(β1→4)-GlcNAc(β1→4)-GlcNAc
Sugar alcohols	32. L-Arabitol	
	33. Xylitol	
Cyclic sugar	34. β-cyclodextrin	$(Glc)_7$
Aromatic alcohols	35. Ferulic acid	4-Hydroxy-3-methoxycinnamic acid
	36. Caffeic acid	3,4-Dihydroxycinnamic acid
	37. Coniferyl alcohol	4-Hydroxy-3-methoxycinnamyl alcohol
	38. Sinapic acid	3,5-Dimethoxy-4-hydroxycinnamic acid
	39. vanillic acid acid	4-Hydroxy-3-methoxybenzoic acid
	40. Coumaric acid	trans-4-Hydroxycinnamic acid

Unless, the L-configuration and the furanose form are denoted, the sugars adopt D-configuration and pyranose forms. *From Megazyme, #From Carbosynth, the remaining substrates are from Sigma. All substrates are of high purity (>90% w/w).

Supplementary Table 3. Apparent kinetic parameters of *Pb*Chi7A-catalysed oxidation of less preferred substrates.

Substrate	K_M (mM)	k_{cat} (s^{-1})	k_{cat}/K_M (s^{-1} M^{-1})
Mannose	417 ± 182	2.80 ± 0.63	7
Mannose*	905 ± 213	0.53 ± 0.05	1
Glucose	231 ± 38	3.50 ± 0.20	15
Glucose*	284 ± 44	0.95 ± 0.05	3
Xylose	333 ± 66	0.20 ± 0.01	1
Galactose	320 ± 55	1.65 ± 0.10	5
Maltose	124 ± 9.1	3.62 ± 0.12	29
Cellobiose	51.0 ± 4.1	3.10 ± 0.07	61
Mannobiose	36.0 ± 13	1.85 ± 0.48	51
Mannotriose	90.5 ± 7.4	2.75 ± 0.16	30

*Dehydrogenase steady-state kinetics of *Pb*Chi7A determined by measuring glucose and mannose oxidation using DCIP the electron acceptor at pH 7.0 and 35 ˚C. The data (n=3 independent experiments) are means ± standard deviations. The oxidase assay was performed at pH 8.0 and 37 ˚C.

Supplementary Table 4. Data collection and refinement statistics (data for the highest-resolution shell are shown in parentheses) for *Fg*CelDH7C and *Fg*Chi7B.

	*Fg*CelDH7C	*Fg*Chi7B
PDB code	6YJI	6YJO
Data collection		
Wavelength	0.9762	0.9677
Resolution range	53.2–1.64 (1.70–1.64)	35.3–2.38 (2.47–2.38)
Space group	$P\ 2_1 2_1 2$	$C\ 2$
Unit cell		
a,b,c (Å), β (˚)	96.54, 187.82, 55.45	111.6, 67.46, 85.08, 116.31
Total reflections	1546975 (91062)	164648 (16216)
Unique reflections	119235 (9035)	22534 (2206)
Multiplicity	13.0 (10.1)	7.3 (7.3)
Completeness (%)	95.55 (73.16)	98.30 (97.35)
Mean I/σ(I)	6.22 (1.07)	10.79 (1.17)
Wilson B-factor	22.67	46.96
R_{merge}	0.1827 (1.547)	0.1385 (1.573)
R_{meas}	0.1901 (1.634)	0.1492 (1.695)
R_{pim}	0.0518 (0.5036)	0.0547 (0.6205)
$CC_{1/2}$	0.996 (0.728)	0.997 (0.454)
CC*	0.999 (0.918)	0.999 (0.79)
Refinement		
Reflections used in refinement	119234 (8942)	22508 (2206)
Reflections used for R_{free}	5909 (425)	1115 (103)
R_{work}	0.1637 (0.2496)	0.2123 (0.3425)
R_{free}	0.2008 (0.2765)	0.2376 (0.3755)
CC(work)	0.966 (0.904)	0.951 (0.703)
CC(free)	0.949 (0.877)	0.942 (0.723)
Number of non-hydrogen atoms	8707	3870
macromolecules	7471	3668
ligands	290	92
solvent	946	110
Protein residues	952	479
RMS(bonds)	0.009	0.003
RMS(angles)	1.00	0.62
Ramachandran favoured (%)	96.31	95.56
Ramachandran allowed (%)	3.59	4.02
Ramachandran outliers (%)	0.11	0.42
Rotamer outliers (%)	0.51	4.21
Clash score	2.48	5.80
Average B-factor	32.36	58.36
macromolecules	31.30	58.87
ligands	45.90	47.75
solvent	36.63	50.12
Number of TLS groups	10	5

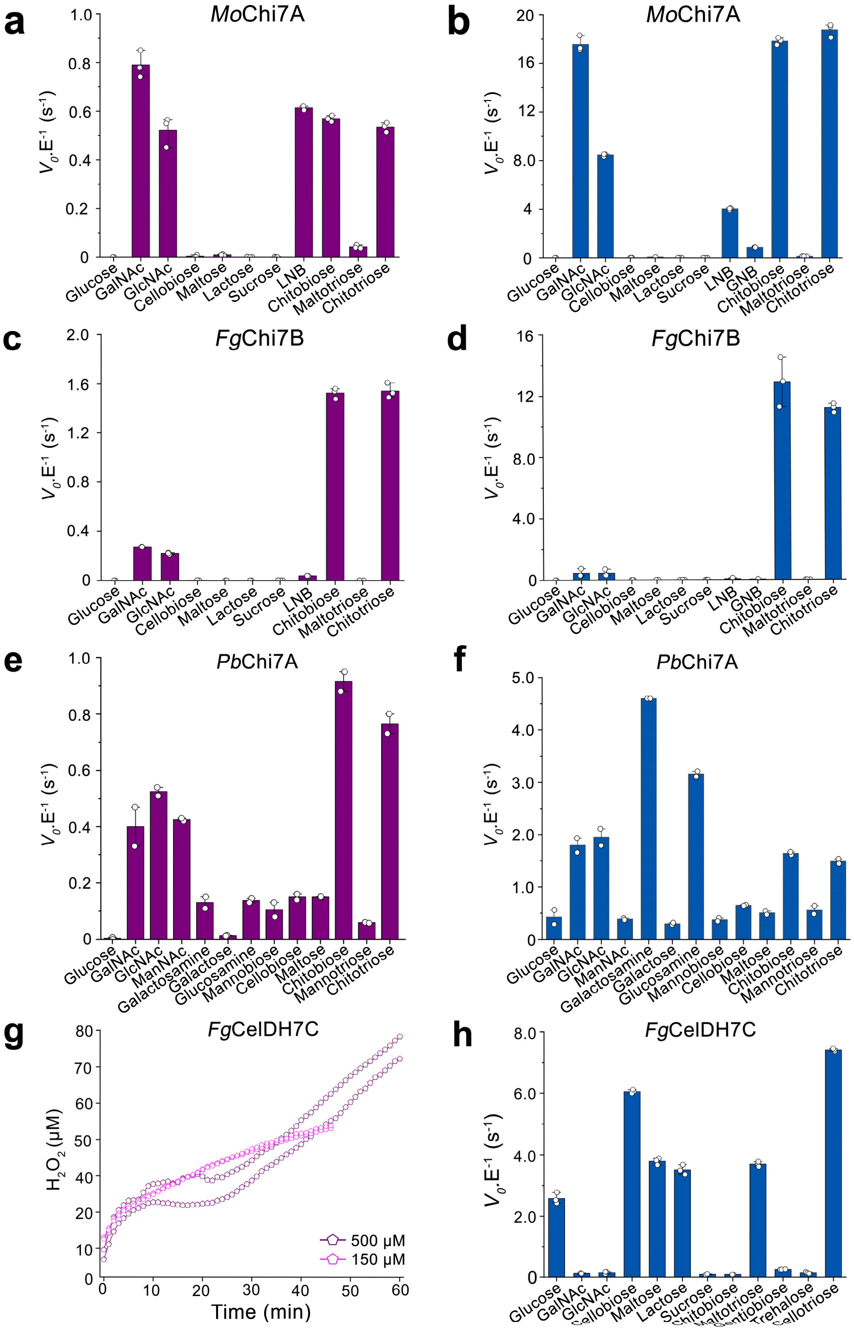

Supplementary Fig. 1. Oxidase and dehydrogenase activity screening of AA7 enzymes. Oxidase (**a, c** and **e)** and dehydrogenase (**b**, **d**, **f and h**) activities shown as normalized rates (V_o·E^{-1}) and the enzyme names are indicated in each panel. **g** The oxidation of cellobiose (20 mM) by 150 and 500 µM of *Fg*CelDH7C monitored using the HRP oxidase assay. The reaction reveals an initial burst followed by linear phase with a lag for the 500 µM reaction. The blank control is in the absence of enzyme (n=2 independent experiments. The screening assay in panels a-f and h was carried out using 0.1 µM of each enzyme. The data (n=3 independent experiments) show the means ± standard deviations, except for panel g, (n=2 independent assays) were performed at each enzyme concentration. Activity on GNB is only analysed in the dehydrogenase assay. Source data are provided as a Source Data file.

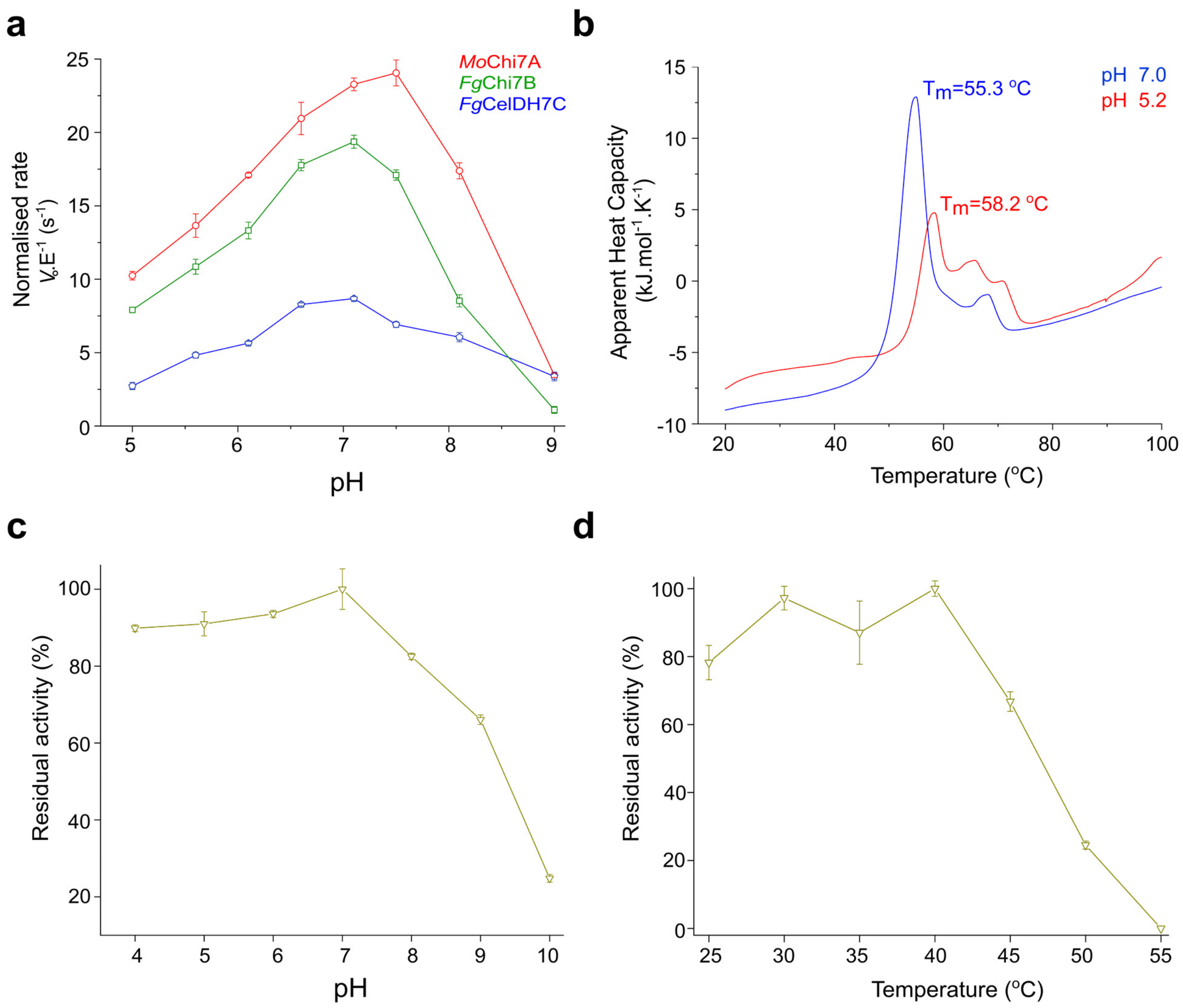

Supplementary Fig. 2. The activity-pH profiles of AA7 enzymes and conformational stability of *Fg*CelDH7C. a The activity-pH profiles of AA7 enzymes as determined using the dehydrogenase assay and DCIP as electron acceptor. The data (n=3 independent experiments) are shown as means ± standard deviations are shown. **b** The differential scanning calorimetry thermograms of the unfolding of 1 mg mL^{-1} *Fg*CelDH7C. The thermograms are from a single measurement (n=1) performed at a scanning rate of 1˚C min^{-1}. **c** and **d** are the normalised residual activities for pH and temperature dependence of *Pb*Chi7A, respectively. Source data are provided as a Source Data file.

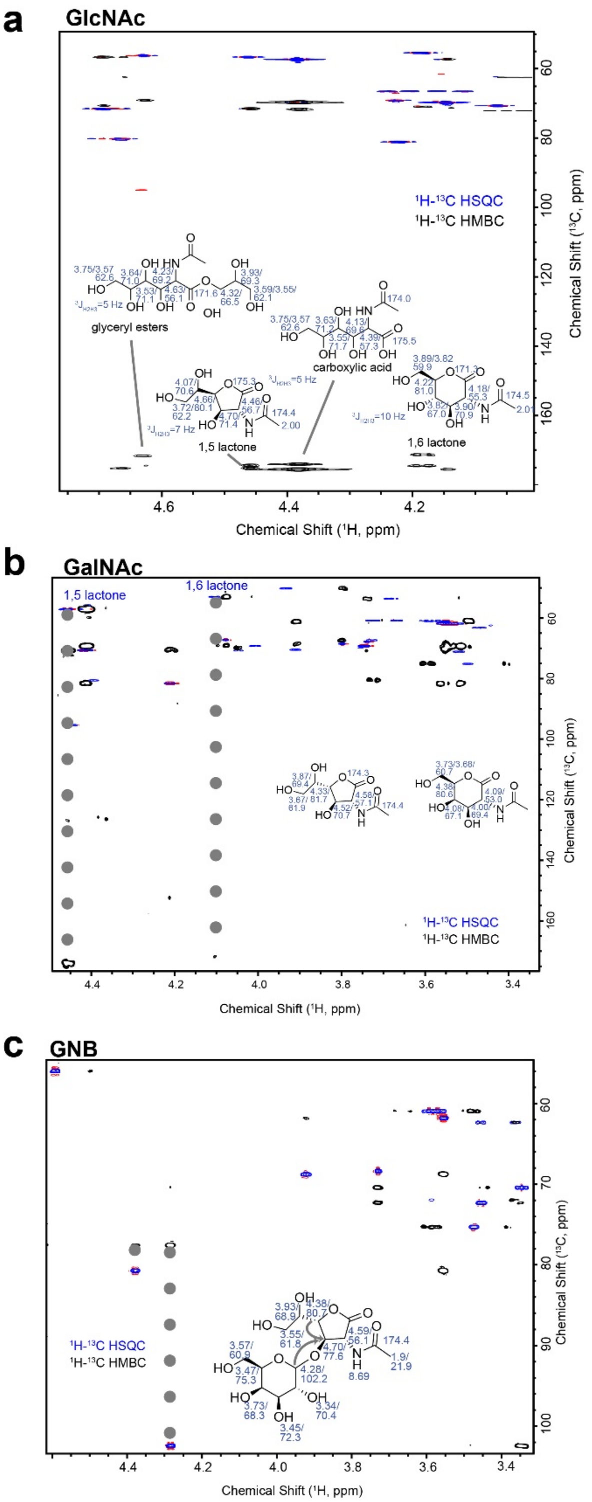

Supplementary Fig. 3. Overlay of ^{1}H-^{13}C HSQC and HMBC NMR spectra for the assignment of intermediates and products formed in the conversion GalNAc, GlcNAc and GNB by *Fg*Chi7B and *Mo*Chi7A. Structural assignment of **a** GalNAc, **b** GlcNAc and **c** GNB as well as their lactone and acid open-form oxidation products are shown alongside the corresponding chemical shift values. The data are from a single experiment (n=1).

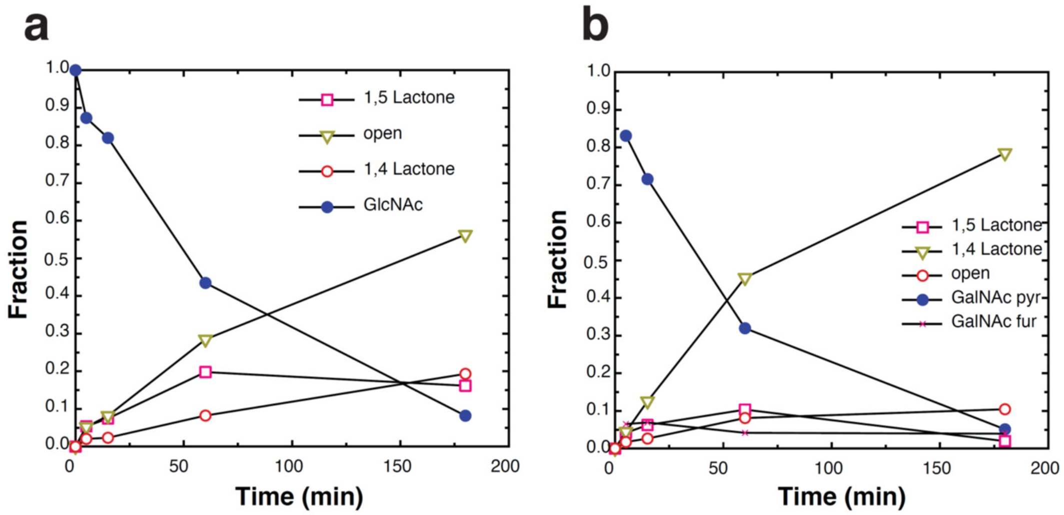

Supplementary Fig. 4. Time course NMR analyses of *Mo*Chi7A oxidation of *N*-acetyl hexosamine monosaccharides. **a** GlcNAc (25 mM) or **b** GalNAc (25 mM), both *Mo*Chi7A (1 µM) in 50 mM NaOAc/AcOH buffer pH 5.2 at 25 ˚C in 0.5 mL NMR samples. The fractions of converted substrate and formed product were approximated using signal areas in a time series of two-dimensional ^{1}H-^{13}C HSQC spectra acquired at 15 ˚C in 0.5 mL NMR samples. The data are from a single experiment (n=1) and source data are provided as a Source Data file.

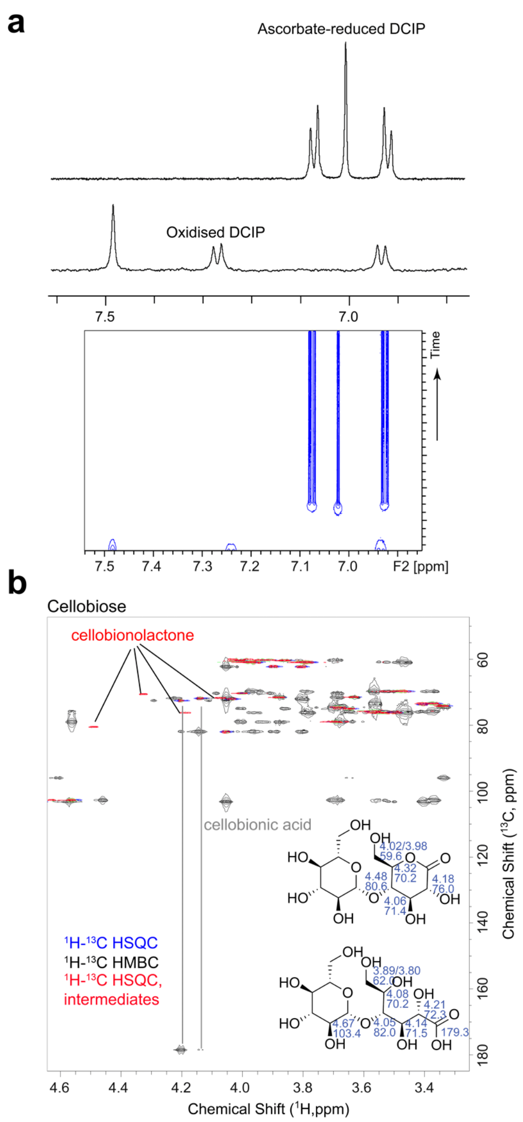

Supplementary Fig. 5 NMR Spectra for the assignment of the electron acceptor DCIP and cellobiose oxidation products. a ^{1}H NMR spectra of ascorbate-reduced DCIP (2 mM in 50 mM NaOAc/OH buffer pH 5.2) and oxidized DCIP and comparison to a time series of ^{1}H NMR spectra recorded during the conversion of cellobiose (2 mM) by *Fg*CelDH7C (0.56 µM) at 25°C in the presence or in the absence of 1.3 mM DCIP. The time series shows the consumption of oxidized DICP and the formation of reduced DCIP. **b** Overlay of ^{1}H-^{13}C HSQC and HMBC NMR spectra yielding the identification of lactone and open-chain oxidation products in the conversion of cellobiose by *Fg*Chi7B, alongside the corresponding chemical shift values. The data are from a single experiment (n=1)

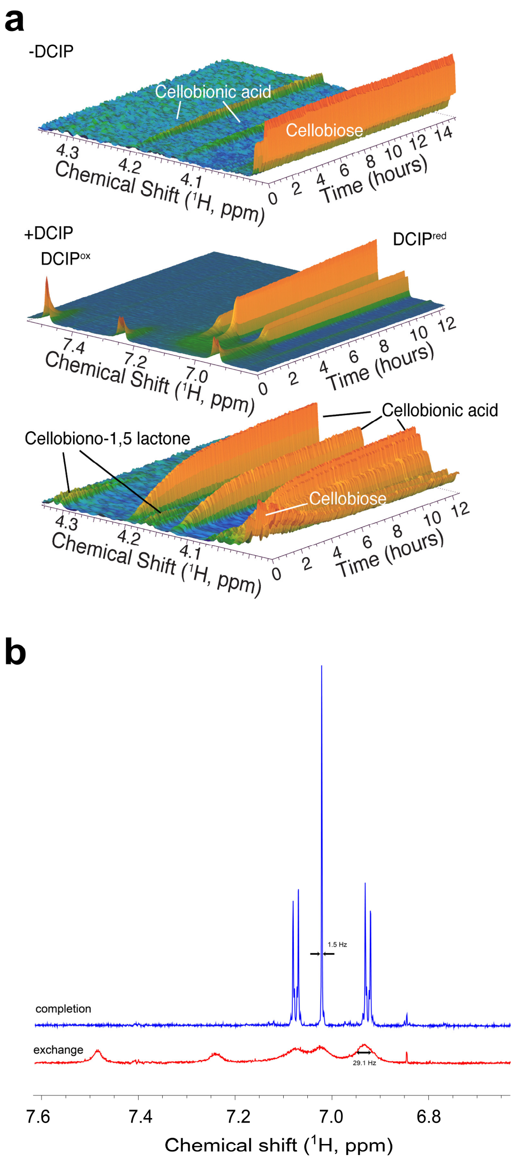

Supplementary Fig. 6. Time course NMR analyses of the oxidation of cellobiose by *Fg*CelDH7C in the presence of and absence of the artificial electron acceptor DCIP. a Time series of ^{1}H NMR experiments for the oxidation of cellobiose (DP2) by *Fg*CelDH7C in the absence (-DCIP, with oxygen as the sole electron acceptor) and in the presence (+DCIP) of 1 mM 2,6-dichlorophenolindophenol. **b** Oxidation of cellobiose (DP2) by *Fg*CelDH7C in the presence of 1.3 mM 2,6-dichlorophenolindophenol (DCIP) electron acceptor. ^{1}H NMR traces after initiation of the reaction (15 minutes time point) and after completion (10 h time point) are shown. In the intermediate-slow exchange regime, the exchange rate constant k_{ex}=$k_1 + k_2$ (with k_1 and k_2 being rate constants for the chemical exchange between distinct states) can be determined from line widths in the presence of exchange and native linewidths upon complete conversion as $k_{ex} = \pi \cdot (w_e - w_0)$. Here, w_e and w_0 are NMR line widths at half height in the presence and in the absence of exchange, respectively. With line widths of 29.1(±0.6) Hz and 1.5(±0.1) Hz upon start of the reaction and upon completion (average line widths for the detected ^{1}H NMR signals of reduced DCIP), the exchange rate constant k_{ex} can be determined to 87 s^{-1}. The data are from one experiment (n=1)

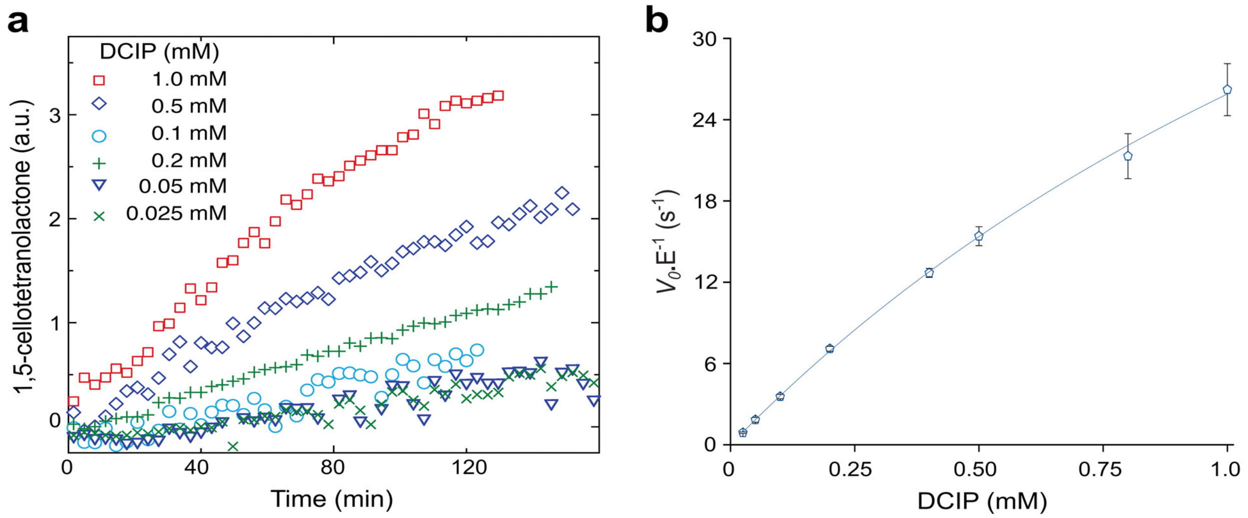

Supplementary Fig. 7. The dependence of cellooligosaccharide oxidation by *Fg*CelDH7C on DCIP. a The oxidation of cellotetraose (6 mM) by *Fg*CelDH7C (0.56 µM) followed by time-resolved *in situ* ^{1}H NMR spectroscopy. **b** The DCIP dependence of the oxidation of cellobiose (20 mM) by *Fg*CelDH7C (0.1 µM) depicted by the Michaelis Menten plot of the oxidation. The high absorbance and poor solubility of DCIP preclude performing the assay at higher DCIP concentration to determine reliable apparent kinetic parameters, but the slight curvature indicates the apparent K_m with respect to DCIP is in the low mM range. The reactions shown in panel a are from a single experiment (n=1) and data in panel b (n=3 independent experiments) are means ± standard deviations. Source data are provided as a Source Data file.

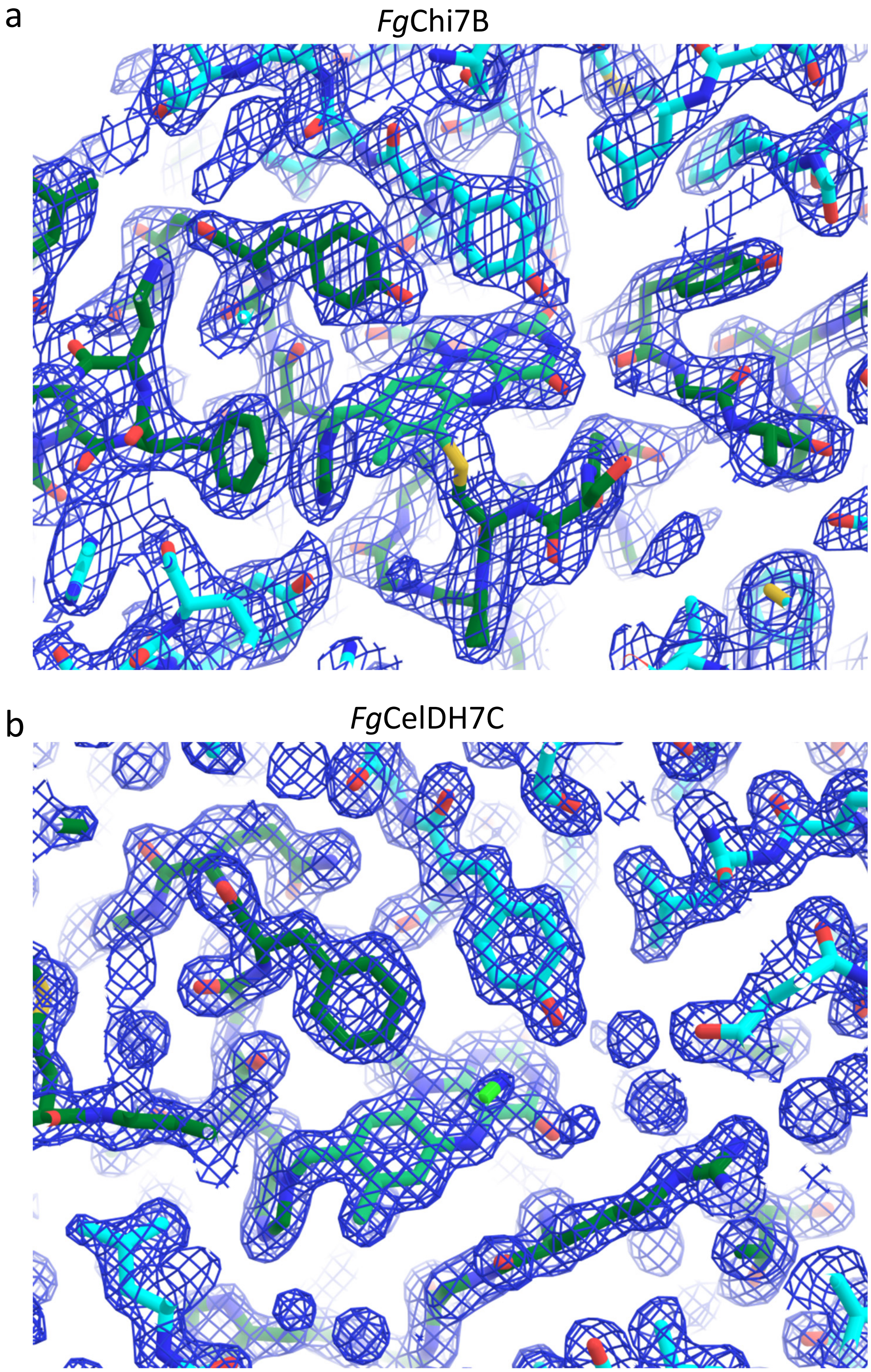

Supplementary Fig. 8. Electron density of the active sites of *Fg*Chi7B and *Fg*CelDH7C. a and **b** show the active site electron densities of *Fg*Chi7B and *Fg*CelDH7C, respectively. The 2mD-Fo maps are contoured at 1.0 σ.

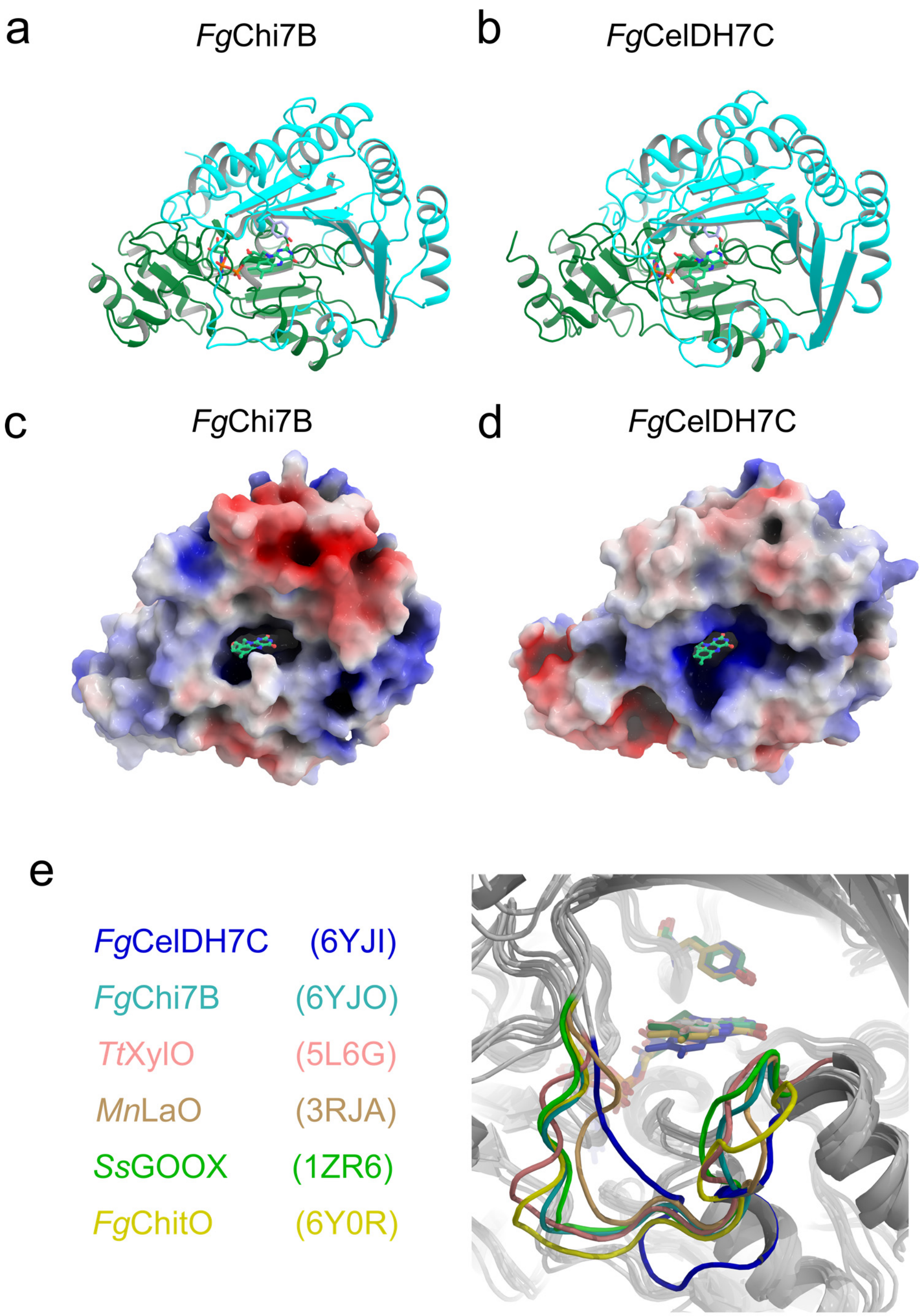

Supplementary Fig. 9. Structural domains and comparisons of AA7 enzymes. a and **b** are the domain organization of *Fg*Chi7B and *Fg*CelDH7C, respectively. The FAD- and substrate-binding domains are in green and cyan, respectively. **c** and **d** show the surface electrostatic potentials of *Fg*Chi7B and *Fg*CelDH7C, respectively as generated from PyMOL. The FAD-cofactor is shown and FAD in both enzymes. **e** Superimposition of structurally characterised oligosaccharide-specific AA7 enzymes. The comparison shows a markedly shorter loop flanking the active site in *Fg*CelDH7C, rendering the active site more open than the compared counterparts.

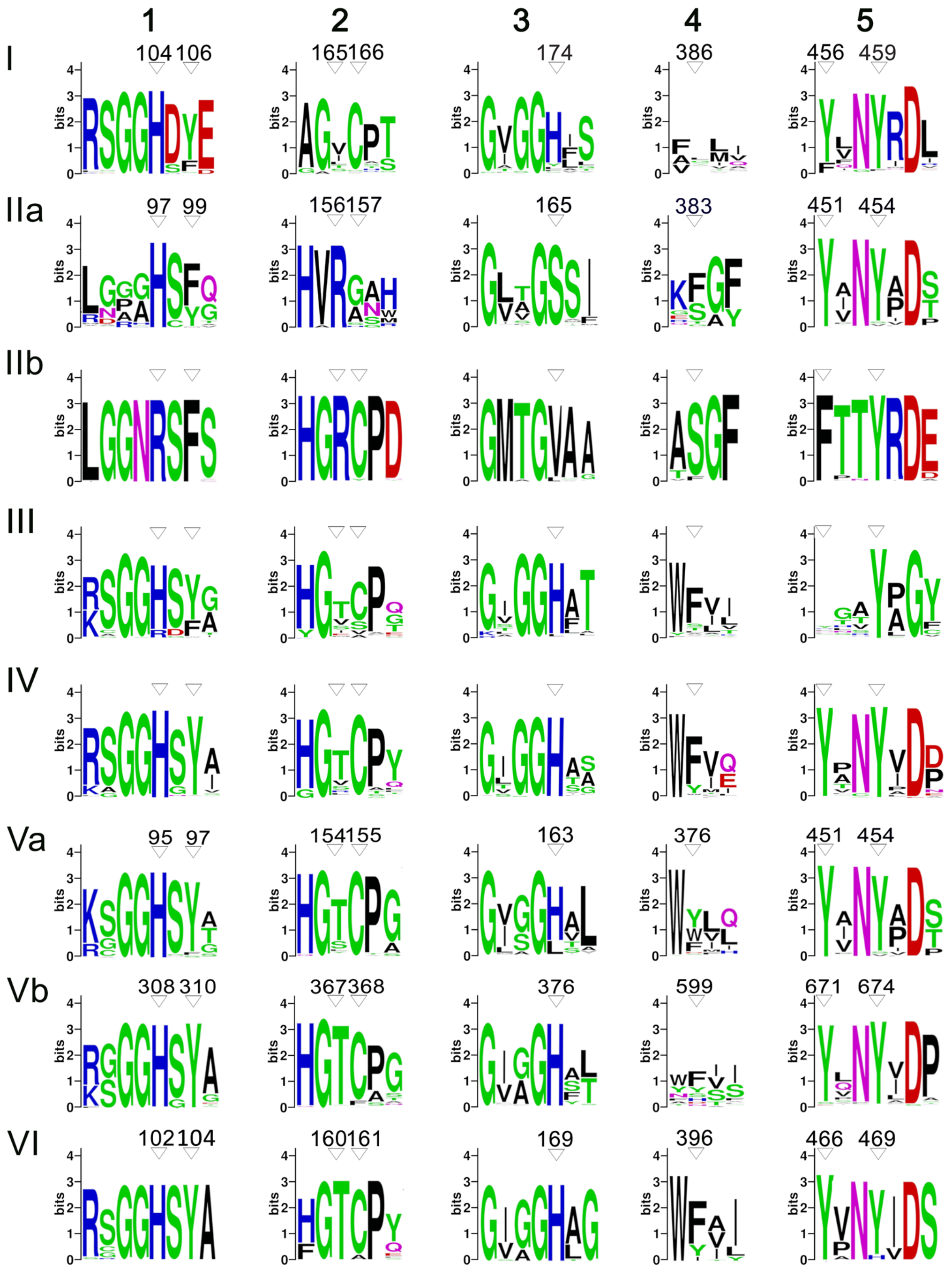

Supplementary Fig. 10. Active site signatures of AA7 clades. The Arabic numerals indicate the conserved active site sequence patches (depicted as sequence logos) as signatures of each clade. Patches 1 and 2 flanking the histidine and cysteine that covalently tether the FAD in canonical AA7 oxidases, respectively; 3, flanks the histidine implicated in O_2 activation in FAD-dependent oxidases; 4, flanks the substrate aromatic stacking platform in carbohydrate-active AA7s; 5 the flanks the catalytic tyrosine. The numbering of key residues is according to biochemically characterised AA7 representing each clade: I, BBE; IIa, *Fg*CelDH7C; Va, *Ss*GOOX; Vb, *Mo*Chi7A; VI, *Pb*Chi7A.

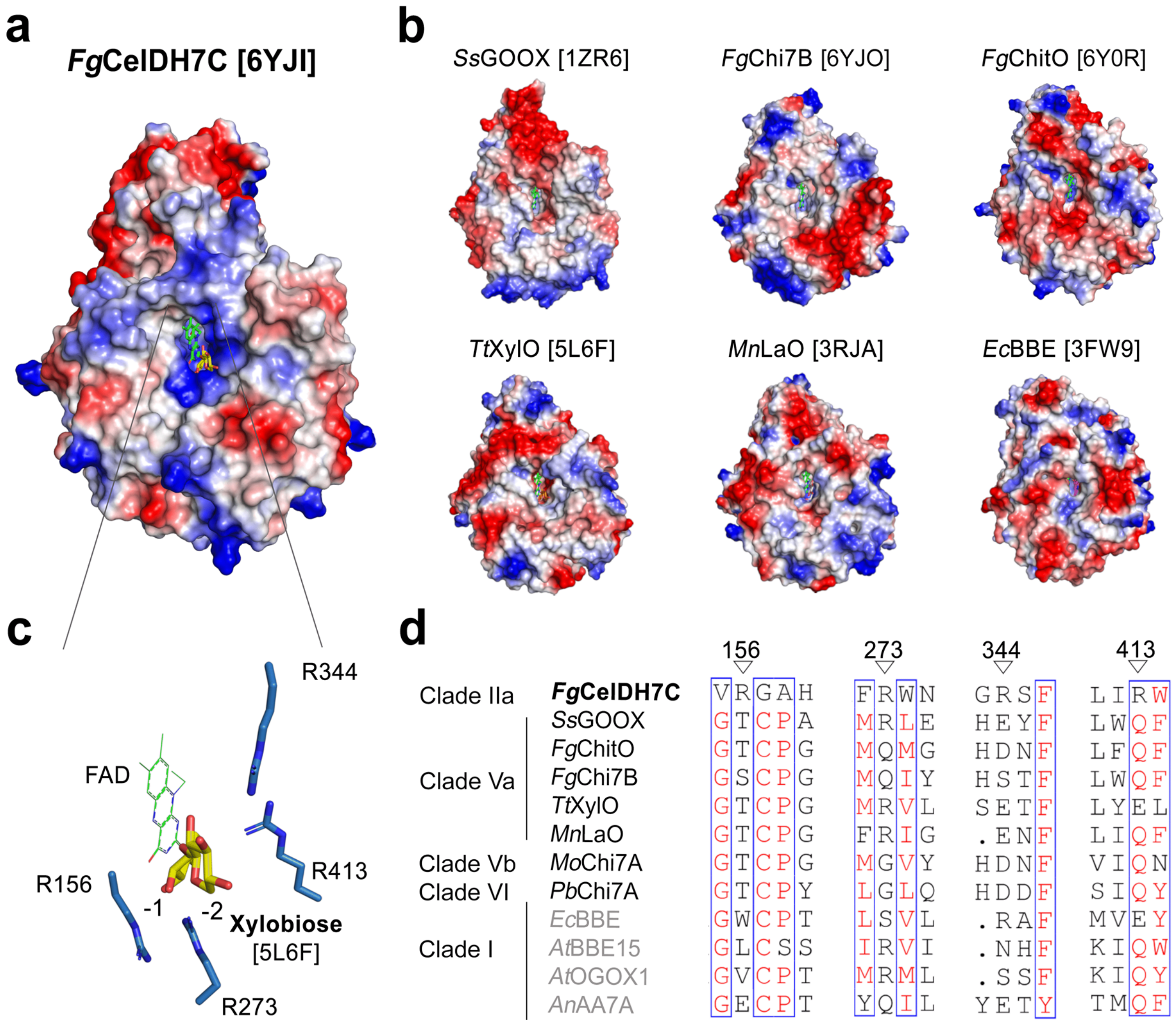

Supplementary Fig. 11. Surface electrostatic comparison of AA7 structures. **a** Surface electrostatic representation (vacuum electrostatics at pH 7.0 calculated in Pymol) of *Fg*CelDH7C, **b** *Fg*Chi7B and the other structurally characterised AA7s. **c** Stick representation of four arginine residues in *Fg*CelDH7C in the vicinity of FAD and substrate binding platform xylobiose is superimposed from *Tt*XylO. **d** Sequence alignment of characterised AA7s in the corresponding regions flanking the four conserved arginine residues in *Fg*CelDH7C and clade II.

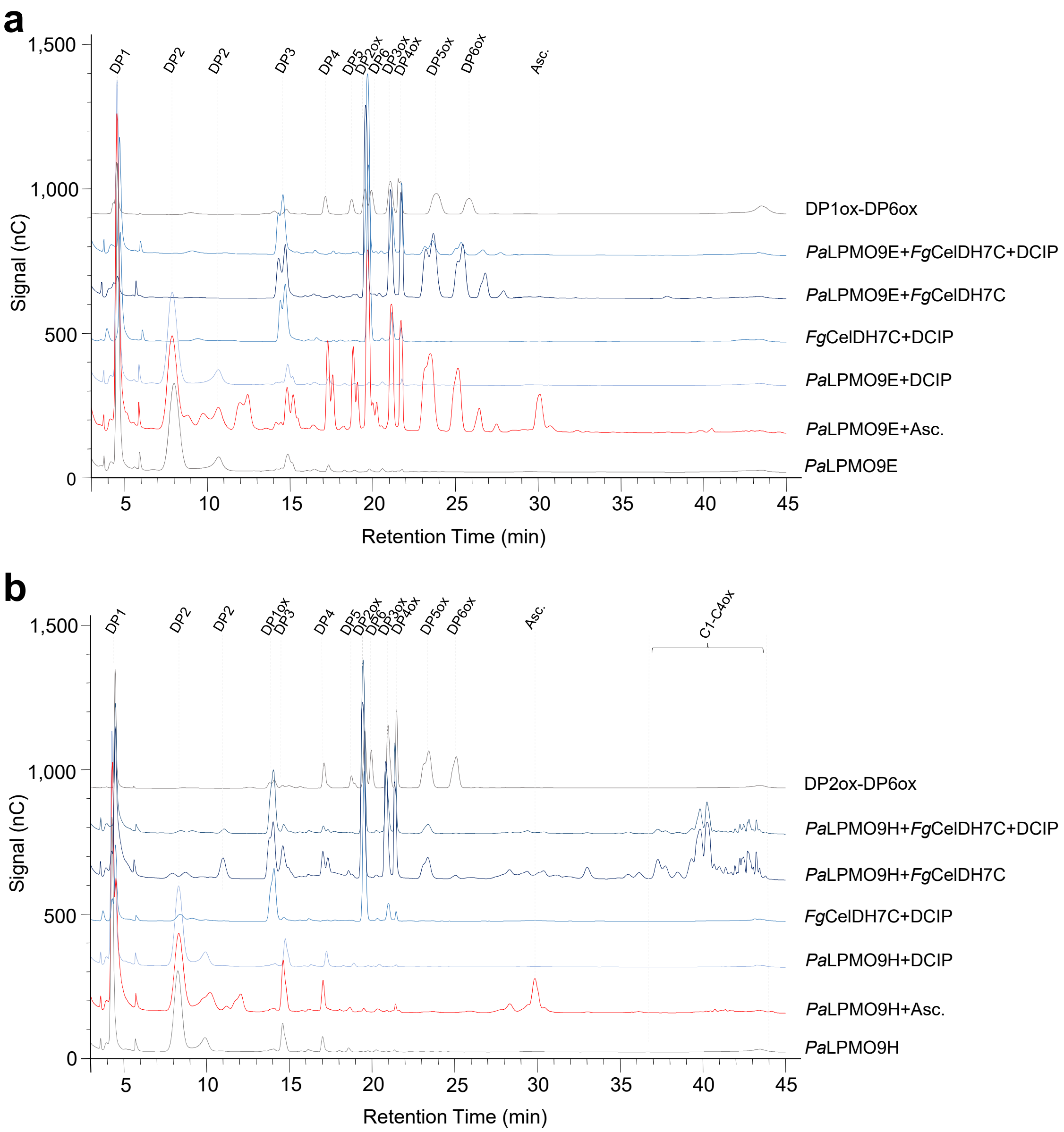

Supplementary Fig. 12. Phosphoric acid swollen cellulose (PASC) degradation assay between *Fg*CelDH7C and either *Pa*LPMO9E or *Pa*LPMO9H. a The assay between *Fg*CelDH7C (0.41 µM) and *Pa*LPMO9E (4 µM) in the absence of additives or in the presence of DCIP. Control experiments of *Pa*LPMO9E alone or in presence of either 0.6 mM DCIP or 1 mM ascorbate were carried out. Reactions were incubated at 30 °C for 18 h and then analysed by HPAEC-PAD. **b** The degradation assay between *Fg*CelDH7C (0.41 µM) and *Pa*LPMO9H (4 µM). Control reactions were prepared using *Pa*LPMO9H with either ascorbate (1 mM), cysteine (1 mM) or DCIP (0.4 mM). All reactions were performed in technical triplicates using 1% (w/v) PASC as a substrate for 24 h. DP refers to the degree of polymerization of native or oxidized (ox) cellooligosaccharides. Reactions were terminated by 10 min incubation at 100 °C and diluted 4-fold with milliQ water prior to HPAEC-PAD analyses. Representative chromatograms are shown (n=3 independent experiments).

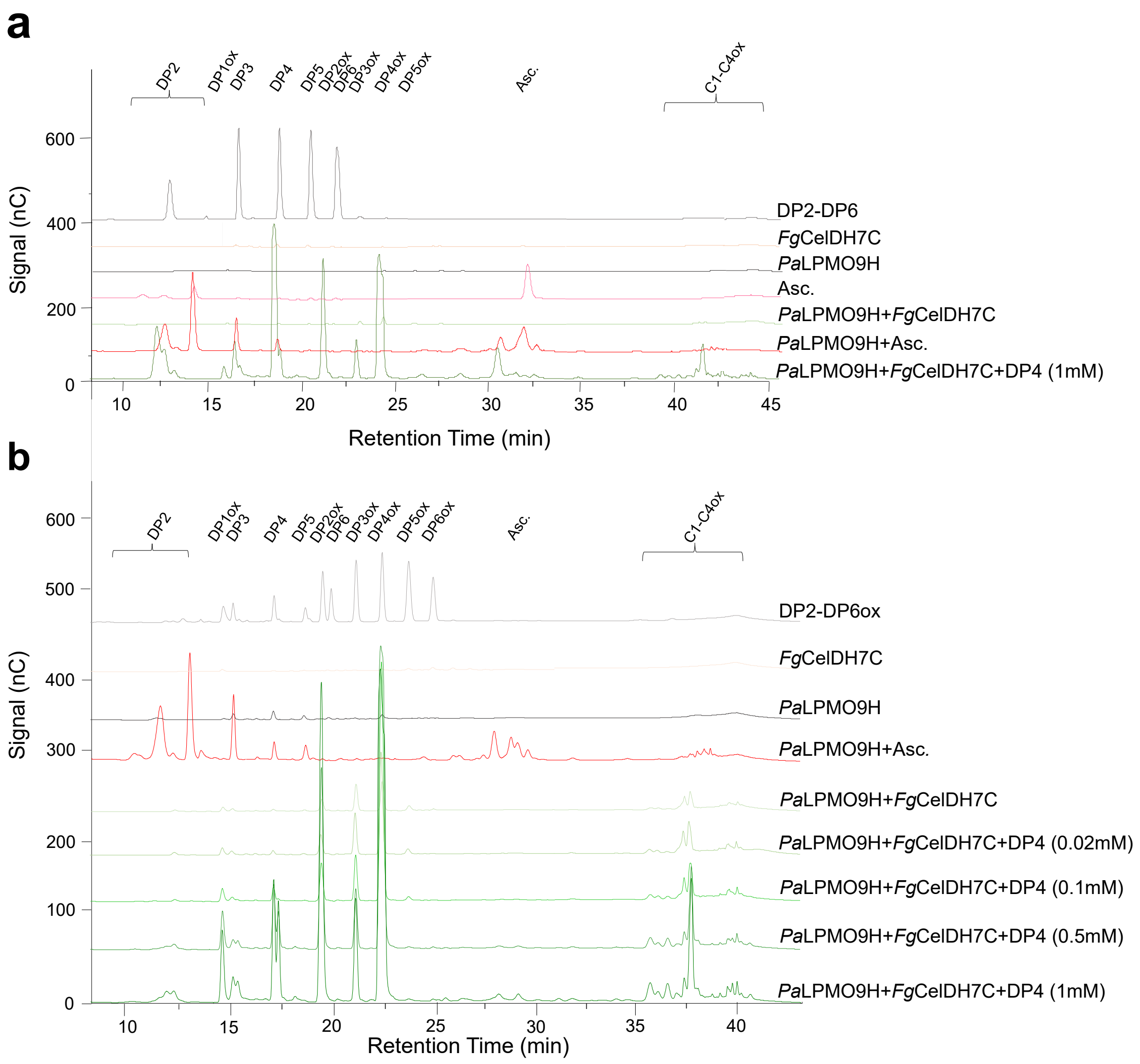

Supplementary Fig. 13. Microcrystalline cellulose (Avicel) degradation with *Fg*CelDH7C-*Pa*LPMO9H. a Assay between *Fg*CelDH7C-*Pa*LPMO9H in the presence of 1 mM cellotetraose (DP4) and 4 h incubation. **b** Effect of added cellotetraose (DP4) concentration (0.02-1 mM) on the synergy between the two enzymes in a after 24 h. The ascorbate concentration was 1 mM. Both synergy were carried out using *Pa*LPMO9H (4 µM) and *Fg*CelDH7C (0.41 µM) incubated with 0.5% (w/v) Avicel at 35°C and subsequently terminated using NaOH (0.1 M) prior to HPAEC-PAD analyses. Representative chromatograms are shown (n=3 independent experiments).

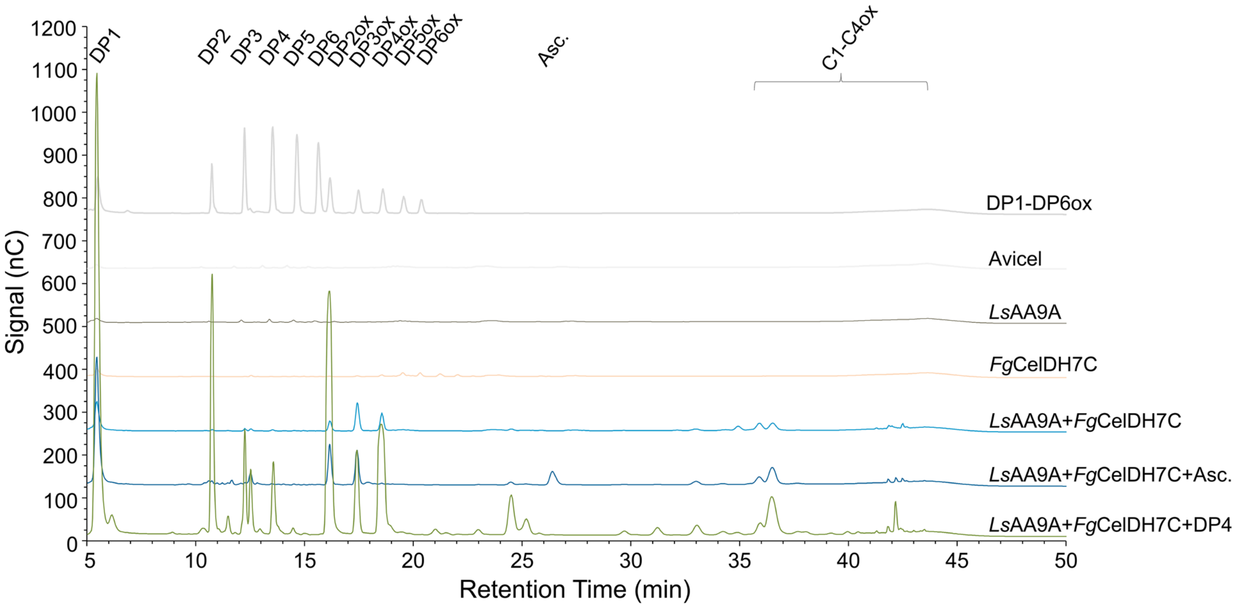

Supplementary Fig. 14. Avicel degradation assay using *Fg*CelDH7C-*Ls*AA9A. The assay between *Fg*CelDH7C and *Ls*AA9A in the presence of 0.8 mM cellotetraose (DP4) and 4 h incubation. The ascorbate concentration was 1 mM. The assay was carried out using *Ls*AA9A (4 µM) and *Fg*CelDH7C (0.2 µM) incubated with 0.5% (w/v) Avicel at 35°C for 24h and subsequently terminated using NaOH (0.1 M) prior to HPAEC-PAD analyses. Source data are provided as a Source Data file. Representative chromatograms (n=3 independent experiments)

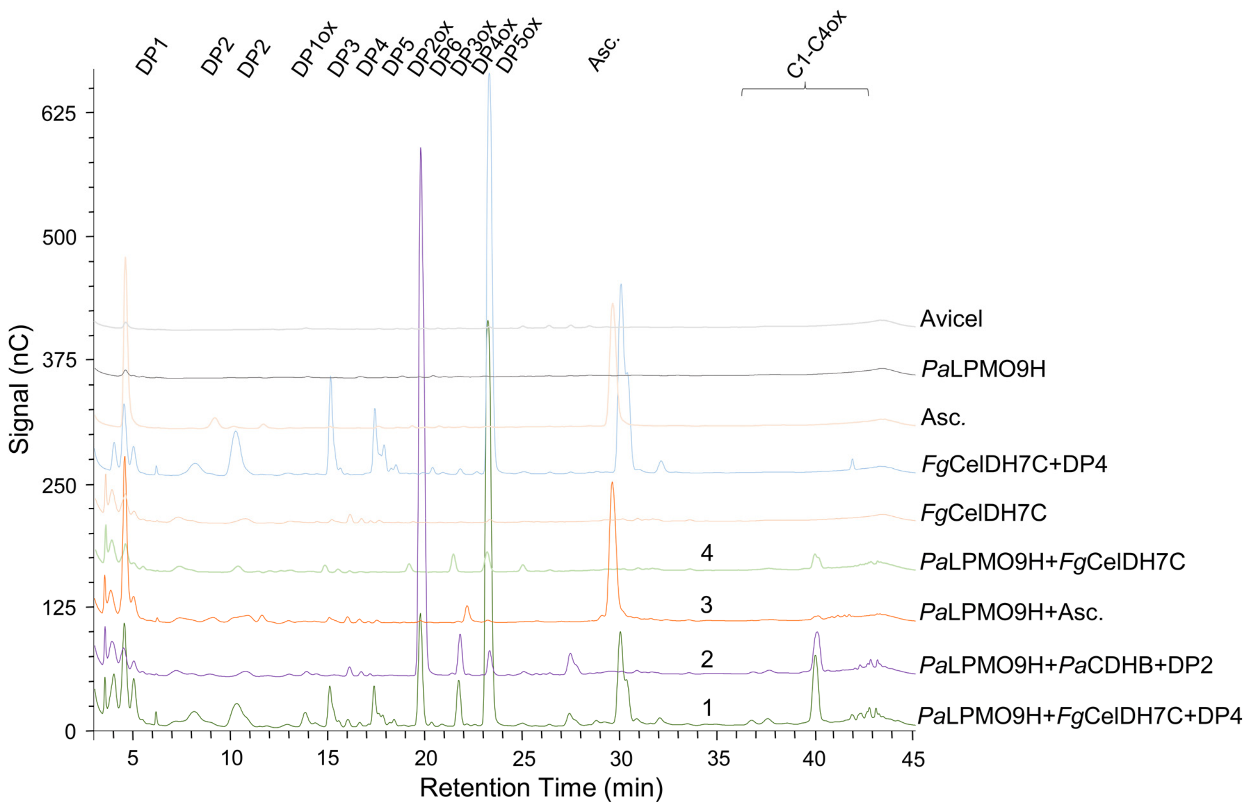

Supplementary Fig. 15. Avicel degradation using *Fg*CelDH7C-*Pa*LPMO9H or *Pa*CDH-*Pa*LPMO9H. Reactions were run on Avicel using the *Fg*CelDH7C and *Pa*LPMO9H enzymes and analyzed using ionic chromatography (HPAEC-PAD). The assays included various combinations of Avicel (5 mg·mL^{-1}), *Fg*CelDH7C (0.4 µM), *Pa*LPMO9H (4 µM µM), *Pa*CDHB (1.2 µM) cellotetraose (DP4, 0.8 mM) and ascorbate (Asc., 1 mM) as indicated in the figure. All reactions were carried out in NaOAc buffer (50 mM, pH 5.2), at 35°C. Native, C1-oxidized and C1-C4 double oxidized cellooligosaccharides are labeled in the chromatograms. The chromatogram numbering is the same as shown in Fig. 4. Source data (n=3 independent experiments) are provided as a Source Data file.

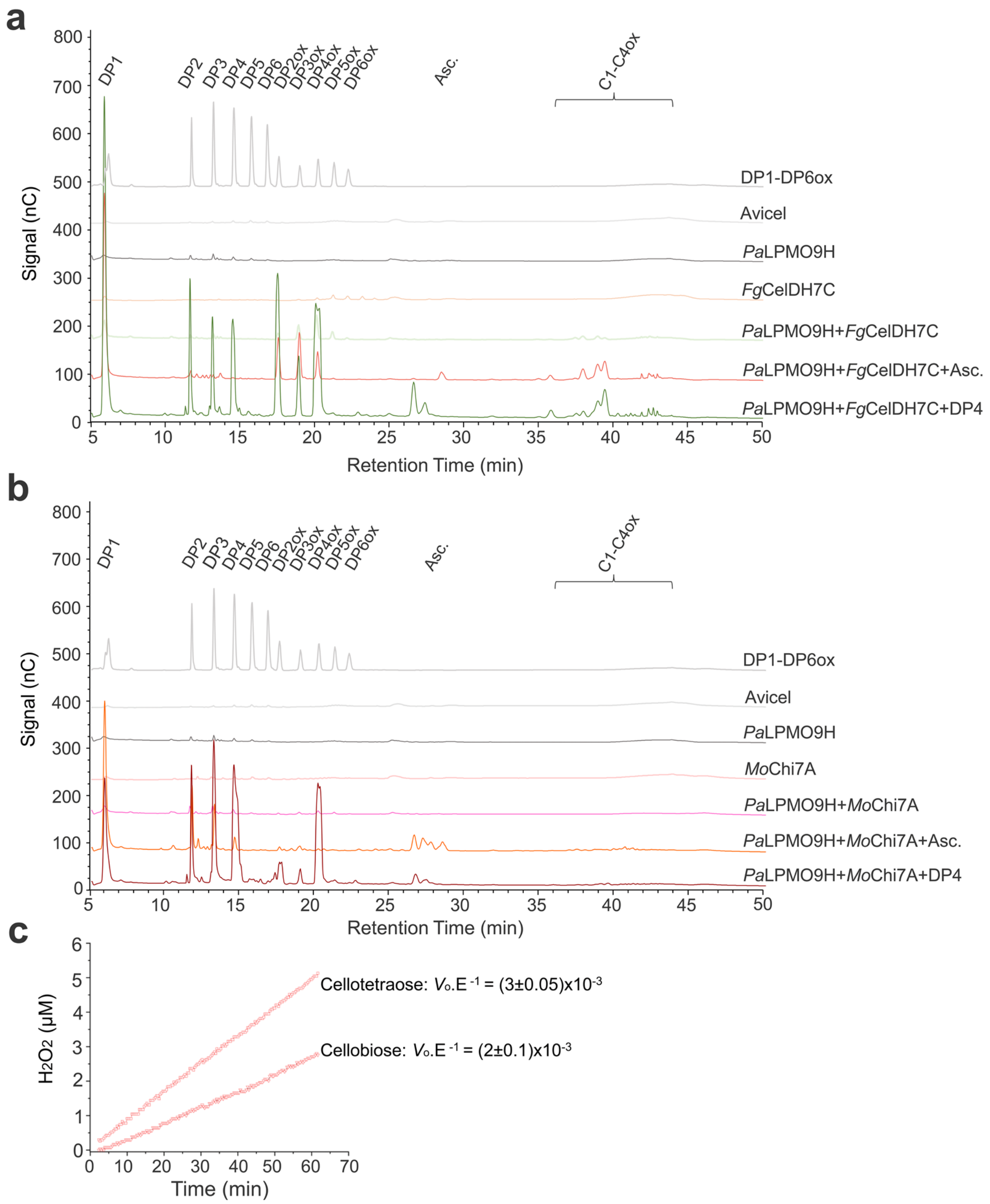

Supplementary Fig. 16. Avicel degradation synergy assay between *Pa*LPMO9H and either *Fg*CelDH7C or *Mo*Chi7A. a The *Fg*CelDH7C-*Pa*LPMO9H synergy assay including control reactions with Avicel, *Fg*CelDH7C, *Pa*LPMO9H, *Pa*LPMO9H+Asc and *Pa*LPMO9H-*Fg*CelDH7C in presence of 0.8 mM cellotetraose (DP4). **b** *Pa*LPMO9H-*Mo*Chi7A synergy assay including controls with Avicel, *Mo*Chi7A, *Pa*LPMO9H, *Pa*LPMO9H+Asc and *Pa*LPMO9H-*Mo*Chi7A in the presence of 0.8 mM cellotetraose. Both assays were carried out using avicel 0.5% (w/v) at 35°C and subsequently terminated using NaOH (0.1 M) prior to analysis by HPAEC-PAD. Native, single oxidized, and double oxidized cello-oligosaccharides are labeled. **c** The oxidase normalized rates of *Mo*Chi7A on cellobiose and cellotetraose are means ± standard deviations. The data in a, b and c are from three independent experiments (n=3).

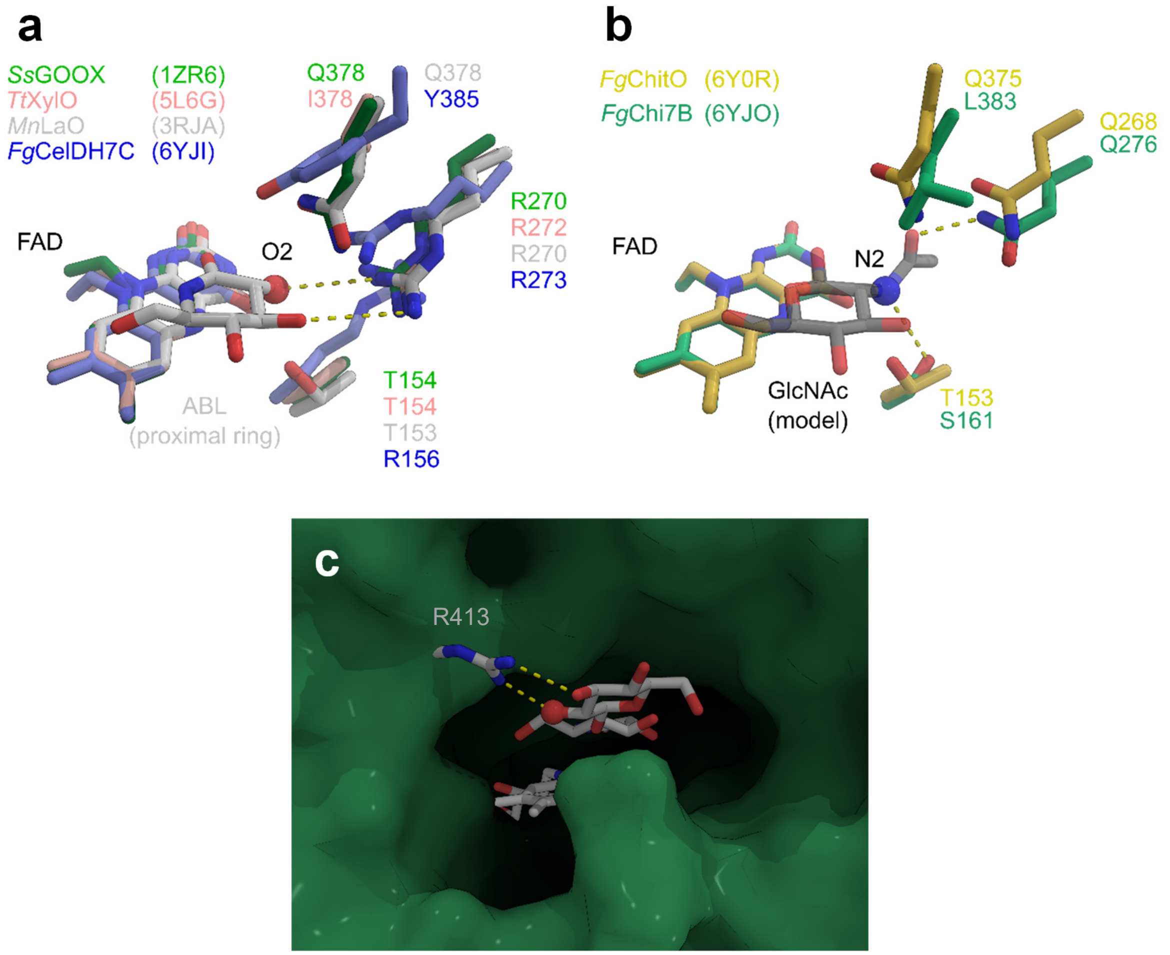

Supplementary Fig. 17. Structural signatures of activity on chitooligosaccharides or other C2 *N*-acetylated substrates. a Structures of AA7 enzymes active on cellooligosaccharides, lactose (*Ss*GOOX, *Mn*LaO, *Fg*CelDH) and xylooligosaccharides (*Tt*XylO), all with a free C2-OH. All these enzymes possess an arginine that recognizes the C2-OH (oxygen as a red sphere) and the C3-OH of the reducing end pyranose (visualized using the proximal ring of the cellobiose analogue ABL (5-amino-5-deoxy-cellobiono-1,5-lactam) in the active site of *Mn*LaO. **b** The two chitooligosaccharide active enzymes with a modelled *N*-acetylglucosamine superimposed on the proximal ring of ABL unit in a. An arginine (as in a) would block the binding of substrates with an *N*-acetyl group. Instead, a smaller residue (commonly a glutamine) provides space and/or the recognition of the *N*-acetyl with a potential hydrogen bond. The *N*-atom of the acetyl group may also be recognised by a small polar residue, *e.g.* S161 in *Fg*Chi7B. **c** The molecular surface of the chitooligosaccharide active *Fg*Chi7B reveals a cavity and available space at the C2-OH (oxygen as red sphere) of the glucosyl penultimate to the oxidation subsite, which is consistent with the tolerance for a C2 *N*-acetyl substitution, *e.g.* in chitobiose. By contrast an arginine residue recognises a non-substituted C2-OH and C3-OH in an analogous manner to the pyranose at the oxidation subsite (see a) in *Mn*LaO. In summary a non-arginine residue is essential for the accommodation of substrates with reducing end C2 *N*-acetyl substitution, while a spacious cavity allows for accommodation of the same substitution at the penultimate position.
