# Supplementary material for: Discovery of fungal oligosaccharide-oxidising flavo-enzymes with previously unknown substrates, redox-activity profiles and interplay with LPMOs
Source: Nat Commun. 2021 Apr 9;12:2132. doi: 10.1038/s41467-021-22372-0 (PMC8035211; doi:10.1038/s41467-021-22372-0)
Supplement: Supplementary file 3 — Reporting Summary [file 41467_2021_22372_MOESM3_ESM.pdf]

nature research

Corresponding author(s): Maher Abou Hachem and Jean-Guy Berrin

Last updated by author(s): 16/02/2021

Reporting Summary

Nature Research wishes to improve the reproducibility of the work that we publish. This form provides structure for consistency and transparency in reporting. For further information on Nature Research policies, see our Editorial Policies and the Editorial Policy Checklist.

Statistics

For all statistical analyses, confirm that the following items are present in the figure legend, table legend, main text, or Methods section.

n/a	Confirmed	
☐	☒	The exact sample size (*n*) for each experimental group/condition, given as a discrete number and unit of measurement
☐	☒	A statement on whether measurements were taken from distinct samples or whether the same sample was measured repeatedly
☒	☐	The statistical test(s) used AND whether they are one- or two-sided<br>*Only common tests should be described solely by name; describe more complex techniques in the Methods section.*
☒	☐	A description of all covariates tested
☒	☐	A description of any assumptions or corrections, such as tests of normality and adjustment for multiple comparisons
☐	☒	A full description of the statistical parameters including central tendency (e.g. means) or other basic estimates (e.g. regression coefficient) AND variation (e.g. standard deviation) or associated estimates of uncertainty (e.g. confidence intervals)
☒	☐	For null hypothesis testing, the test statistic (e.g. *F*, *t*, *r*) with confidence intervals, effect sizes, degrees of freedom and *P* value noted<br>*Give P values as exact values whenever suitable.*
☒	☐	For Bayesian analysis, information on the choice of priors and Markov chain Monte Carlo settings
☒	☐	For hierarchical and complex designs, identification of the appropriate level for tests and full reporting of outcomes
☒	☐	Estimates of effect sizes (e.g. Cohen's *d*, Pearson's *r*), indicating how they were calculated

Our web collection on statistics for biologists contains articles on many of the points above.

Software and code

Policy information about availability of computer code

Data collection	The Topspin 3.5 pl6 has been used for the analysis of the NMR data. The MXCuBE v3 was used at BioMAX/MAX IV, while the MXCuBE v2 was used at MASSIF-3/ESRF.
Data analysis	Origin (v9.55 & v18) has been used for graphics. The Chromeleon 6.80 SR10 software has been used to analyse HPAEC data. The Topspin 3.5 pl6 has been used for the analysis of the NMR data. The following programs have been used for the structural determination: Phaser 3.24.1, for molecular replacement, The PyMol software version 2.2.0 has been used for the structural analyses and molecular rendering. PHENIX.autobuild and PHENIX.refine as well as COOT v0.9 for model building and refinement. The manual model was analysed using MolProbity. The Protparam tool was used for protein sequence analysis. SignalP 5.0 was used for signal peptide prediction. BlastP was used for protein sequence search and retrieval. MAFFT was used for sequence alignment and Gblock0.91b was used for curating the sequence alignment. The phylogenetic analysis was performed using NGphylogeny and rendered using iTOL. The visualization of amino acid conservation was made using WebLogo.

For manuscripts utilizing custom algorithms or software that are central to the research but not yet described in published literature, software must be made available to editors and reviewers. We strongly encourage code deposition in a community repository (e.g. GitHub). See the Nature Research guidelines for submitting code & software for further information.

Data

Policy information about availability of data

All manuscripts must include a data availability statement. This statement should provide the following information, where applicable:

- Accession codes, unique identifiers, or web links for publicly available datasets
- A list of figures that have associated raw data
- A description of any restrictions on data availability

The atomic coordinates of FgCelDH7C and FgChi7B have been deposited in the Protein Data Bank (https://www.rcsb.org) under the PDB accessions 6YJI and 6YJO, respectively (see also Supplementary Table 4). The GenPept accession IDs of the enzymes characterised in the study are XP_660252, XP_011319890, CEF79461 XP_003717634, RDX44700.1 (see Supplementary Table 1). All the data are available from the corresponding authors upon request. Source data are provided with this paper as supplementary file.

Field-specific reporting

Please select the one below that is the best fit for your research. If you are not sure, read the appropriate sections before making your selection.

- [x] Life sciences
- [ ] Behavioural & social sciences
- [ ] Ecological, evolutionary & environmental sciences

For a reference copy of the document with all sections, see nature.com/documents/nr-reporting-summary-flat.pdf

Life sciences study design

All studies must disclose on these points even when the disclosure is negative.

Sample size	Most experiments were performed in triplicates. The NMR and the EPR experiments were carried out once, as the informational content of these experiments is deemed robust and sufficient.
Data exclusions	No data were excluded from the study.
Replication	The experiments were performed in triplicates unless otherwise stated, e.g. the NMR data.
Randomization	The technical aspects of the methods and the small sample size makes randomization less relevant.
Blinding	Not relevant, as this study does not include experiments with obvious risk of bias if the experiments are not blinded.

Reporting for specific materials, systems and methods

We require information from authors about some types of materials, experimental systems and methods used in many studies. Here, indicate whether each material, system or method listed is relevant to your study. If you are not sure if a list item applies to your research, read the appropriate section before selecting a response.

Materials & experimental systems

n/a	Involved in the study	
☒	☐	Antibodies
☒	☐	Eukaryotic cell lines
☒	☐	Palaeontology and archaeology
☒	☐	Animals and other organisms
☒	☐	Human research participants
☒	☐	Clinical data
☒	☐	Dual use research of concern

Methods

n/a	Involved in the study	
☒	☐	ChIP-seq
☒	☐	Flow cytometry
☒	☐	MRI-based neuroimaging
